# The Association Between the STOP-Bang Score and the Integrated Pulmonary Index in Patients Undergoing Endobronchial Ultrasound with Sedation: The STOP OSA-IPI Cohort Study

**DOI:** 10.3390/medicina62061034

**Published:** 2026-05-26

**Authors:** Umran Ozden Sertcelik, Mustafa Turker, Ahmet Sertcelik, Ebru Sengul Parlak, Habibe Hezer, Kubra Gungor, Mithat Temizer, Seyhan Yagar, Aysegul Karalezli

**Affiliations:** 1 Department of Chest Diseases, Faculty of Medicine, Ankara Yıldırım Beyazıt University, 06800 Ankara, Türkiye; aysegulkaralezli@aybu.edu.tr; 2 Department of Chest Diseases, Ankara Bilkent City Hospital, 06800 Ankara, Türkiye; ebrusengul.serefparlak@sbu.edu.tr (E.S.P.); hoflaz@yahoo.com (H.H.); kubraturkels@gmail.com (K.G.); 3 Department of Anesthesiology and Reanimation, Ankara Bilkent City Hospital, 06800 Ankara, Türkiye; m95turker@gmail.com (M.T.); seyhan.yagar@sbu.edu.tr (S.Y.); 4 Department of Infectious Diseases and Clinical Microbiology, Division of Epidemiology, Faculty of Medicine, Ankara Yıldırım Beyazıt University, 06800 Ankara, Türkiye; ahmetsertcelik@aybu.edu.tr; 5 Department of Infectious Diseases and Clinical Microbiology, Ankara Bilkent City Hospital, 06800 Ankara, Türkiye; 6 Department of Chest Diseases, Faculty of Gulhane Medicine, Health Sciences University, 06100 Ankara, Türkiye; 7 Department of Public Health Division of Epidemiology, Faculty of Medicine, Manisa Celal Bayar University, 45030 Manisa, Türkiye; mithat.temizer@cbu.edu.tr; 8 Department of Anesthesiology and Reanimation, Faculty of Gulhane Medicine, Health Sciences University, 06100 Ankara, Türkiye

**Keywords:** bronchoscopy, capnography, deep sedation, endosonography, obstructive sleep apnea, oxygen deficiency

## Abstract

*Background and Perspectives*: Obstructive sleep apnea (OSA) is a prevalent condition associated with increased perioperative risks. Endobronchial ultrasound (EBUS), a diagnostic and staging procedure requiring deep sedation, may pose additional risks for patients at high risk of OSA. The Integrated Pulmonary Index (IPI), derived from capnography and vital signs, offers a single numerical value reflecting respiratory status. This study aimed to assess the association between high OSA risk and adverse events using the IPI during EBUS under sedation. *Materials and Methods*: This prospective cohort study included 65 patients undergoing EBUS with sedation between December 2024 and April 2025 at a tertiary referral center. STOP-Bang questionnaire scores were used to stratify patients into high- (≥3) and low-risk (<3) OSA groups. During the procedure, IPI, oxygen saturation, end-tidal carbon dioxide, respiratory rate, and hemodynamic parameters were recorded at multiple time points. Hypoxemia, hypoventilation, and apnea were defined using standard thresholds. Logistic regression and Generalized Linear Mixed Models (GLMM) were applied to examine associations between OSA risk and respiratory outcomes. *Results*: Forty-three patients (66.2%) were classified as high risk for OSA. Patients with high STOP-Bang scores were older and had higher BMI, comorbidity rates, and ASA scores (all *p* < 0.05). IPI values were lowest between 5 and 10 min, accompanied by more frequent interventions. Logistic regression showed no significant association between STOP-Bang scores and low IPI or hypoxemia. GLMM analysis also indicated no significant association between high OSA risk and low IPI (OR = 1.02; 95% CI = 0.36–2.86; *p* = 0.974). Hypoxemia was nearly threefold higher in high-risk patients, though not statistically significant (*p* = 0.080). *Conclusions*: Although no statistically significant association was identified between high OSA risk and adverse respiratory events, GLMM analyses revealed that patients with high STOP-Bang scores demonstrated approximately three times higher odds of developing hypoxemia (OR = 2.76; 95% CI = 0.99–7.66; *p* = 0.052), a result that approached statistical significance. The present findings do not support the routine use of IPI-based monitoring in this setting, and further adequately powered studies are warranted. The early procedural period (5–10 min) is critical for hypoxemia and respiratory compromise.

## 1. Introduction

Obstructive sleep apnea (OSA) is characterized by recurrent complete or partial upper airway obstruction during sleep [1,2]. It is estimated that approximately 1 billion adults worldwide are affected by OSA [3]. A cohort study of 1525 adults revealed that 61% of women and 84% of men had OSA [4]. According to a study involving a representative sample of 5021 participants in Türkiye, the estimated prevalence of OSA was reported to be 14% [5]. Polysomnography performed overnight is the gold standard for diagnosing OSA. However, this method is time-consuming, labor-intensive, and costly [6,7]. Although OSA patients have a higher risk of developing perioperative cardiac and pulmonary complications, approximately 60% of patients with moderate to severe OSA are not diagnosed prior to surgery [8,9]. OSA patients may experience obstructive episodes during procedures under sedation [10,11].

Endobronchial ultrasound (EBUS) is a procedure that allows for the examination and sampling of both the airways and structures adjacent to the airways, as well as mediastinal lymph nodes, using a probe inserted through the working channel of a bronchoscope after entering the airways [12]. Sedation is necessary for patient comfort and procedural quality during EBUS. It is an important component of the procedure as it improves patient comfort and may increase the diagnostic accessibility [13]. The procedure duration may extend depending on the number of stations where EBUS-transbronchial needle aspiration is performed and the patient’s condition. Therefore, the need for and duration of sedation and analgesia in patients undergoing EBUS is higher compared to other procedures [14].

Studies have shown that monitoring the procedure with capnography may help reduce hypoxic events (oxygen saturation (SpO_2_) < 90% for more than 15 s) and severe hypoxia (SpO_2_ < 85%) [14,15]. Therefore, global guidelines suggest that capnography may provide additional safety during deep sedation [15]. The Integrated Pulmonary Index (IPI), which is measured and calculated using a specific capnograph, is an algorithm that provides a combined assessment of four parameters: heart rate, respiratory rate, SpO_2_, and end-tidal carbon dioxide (etCO_2_). This index offers a simplified interpretation of the patient’s respiratory status with a single value [16].

Adverse events and interventions are more common during the procedure, especially in patients at high risk of OSA [14,17,18]. However, the relationship between the IPI and adverse events in patients at risk of OSA during EBUS has not been sufficiently defined. We consider that hypoxia and hypoventilation events may be more common in individuals with clinically undiagnosed OSA. While it is known that the risk of these adverse events is higher in OSA cases, information on patients undergoing EBUS is limited.

We hypothesized that patients with high OSA risk (STOP-Bang score ≥ 3) would demonstrate lower IPI values and higher rates of hypoxemia and hypoventilation during EBUS under sedation compared to patients at low OSA risk (STOP-Bang score < 3). The primary outcome was the association between high OSA risk and low IPI (defined as IPI < 7 at any procedural time point); the secondary outcome was hypoxemia.

## 2. Materials and Methods

**Figure 1:**
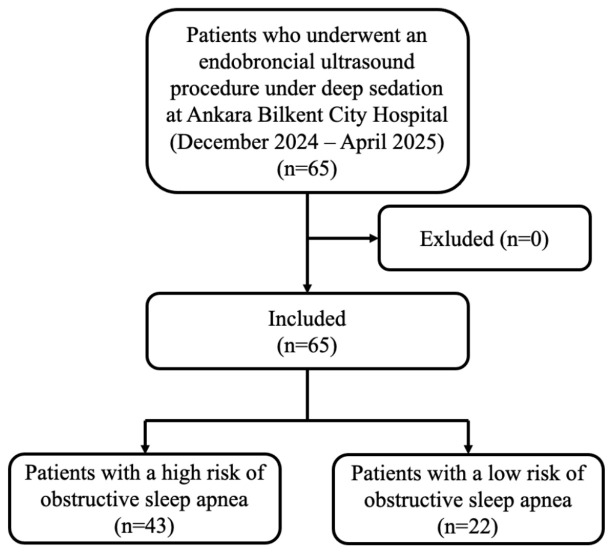
Flow chart of the study.

This prospective cohort study included patients who underwent EBUS with sedation between December 2024 and April 2025 at Ankara Bilkent City Hospital, Ankara Yıldırım Beyazıt University Bronchoscopy Unit, a 4050-bed tertiary care teaching hospital and one of Türkiye’s largest referral centers [19]. Patients with an OSA diagnosis, American Society of Anesthesiologists (ASA) physical status > 3, tracheostomy tube, basal pulse oximeter SpO_2_ < 90%, respiratory rate > 20/min, peripheral vascular disease, psychotic and antidepressant medication use, and inability to provide informed consent were planned to be excluded from the study. No patients met the exclusion criteria during the study period (Figure 1). This study was approved by the Ethics Review Committee of Ankara Bilkent City Hospital. All participants signed a written informed consent form, and the study was conducted in accordance with the principles of the Helsinki Declaration.

### 2.1. Patients and Data

All 65 patients who underwent EBUS between the specified dates were included in the study. The patients’ age, gender, comorbidities, ASA risk score, Mallampati score, and smoking status were recorded through face-to-face interviews with the patients prior to the procedure. A self-administered STOP-Bang questionnaire was applied under the researchers’ supervision. The STOP-Bang score was calculated after the procedure. The researchers and the procedure team were blinded to the STOP-Bang results during the procedure. The respiratory rate, etCO_2_ value, and IPI obtained from the capnography device were recorded at specified times. During the procedure, hypoxemia, hypoventilation, and apnea conditions, along with the IPI value, were recorded at specified times, along with all interventions performed on the patients and the oxygen support provided. After the procedure, the procedure duration and total doses of medications administered to the patient for sedation were recorded. Routine intraoperative saturation, heart rate, and blood pressure values were recorded at specified times. The researchers recorded all relevant data in a specific data form prepared for the study.

### 2.2. Definitions and Interventions

An SpO_2_ < 90% attack lasting longer than 15 s was defined as hypoxemia, an etCO_2_ > 50 mmHg lasting longer than 15 s was defined as hypoventilation, and a cessation of breathing lasting longer than 30 s was defined as apnea [14,17]. In this study, hypoventilation was operationally defined for capnographic monitoring purposes as an etCO_2_ value exceeding 50 mmHg for more than 15 s. This definition was used as a monitoring-based surrogate and does not represent the American Academy of Sleep Medicine (AASM) physiological definition of hypoventilation. Hypoxemia was operationally defined as an SpO_2_ value < 90% persisting for more than 15 s, based on continuous pulse oximetry monitoring. This definition was used as a pragmatic monitoring surrogate and does not correspond to AASM polysomnographic hypoxemia or desaturation criteria.

Patients included in the study were defined as having a high risk of OSA if their STOP-Bang Score was 3 or higher, and a low risk of OSA if their STOP-Bang Score was below 3 [7]. An IPI value of 7 or higher was defined as normal, and a value below 7 was defined as low IPI [20]. Height and body weight were measured using standardized procedures. Height was measured to the nearest 0.1 cm using a stadiometer (Model GL-150, Densi, Istanbul, Türkiye) with participants standing barefoot in an upright position. Body weight was measured to the nearest 0.1 kg using a calibrated digital scale (Model GL-150, Densi), with participants wearing light clothing and no shoes. The body mass index (BMI) was calculated by dividing the patient’s weight by the square of their height [21]. The neck circumference was measured using a flexible tape at the level of the thyroid cartilage, with the patient in a seated or standing position and the head in a neutral position [22]. The neck–height ratio was calculated by dividing the circumference of the neck by the height. Smoking pack-years were calculated by multiplying the number of years smoked by the number of cigarette packs consumed daily. Blood pressure was monitored using a validated automated device (Model B40, General Electric Medical Systems Information Technologies GmbH, Freiburg, Germany). The mean arterial pressure was obtained by dividing the sum of twice the systolic blood pressure and diastolic blood pressure by three. The interventions applied to patients during the procedure were: patient alerting (pain stimulus), discontinuation of or reduction in sedatives (sedation cessation), increasing O_2_ delivery up to 15 L/min (increasing fraction of inspired oxygen (FiO_2_), chin lift or chin thrust maneuver (jaw thrust), airway application, oropharyngeal aspiration, interrupting the procedure, terminating the procedure, and methylprednisolone administration.

### 2.3. OSA Screening

The STOP-Bang questionnaire was used as the OSA screening tool. The STOP-Bang questionnaire is calculated based on snoring, fatigue, witnessed apnea, hypertension, BMI, age, neck circumference, and gender information. The STOP-Bang questionnaire was developed to screen for undiagnosed OSA patients in the preoperative setting and has been validated [7,23]. The validity of the Turkish version has been demonstrated by Acar et al. [24]. The questionnaire consists of a total of eight ‘yes’ or ‘no’ questions (snoring, fatigue, witnessed apnea, hypertension, BMI > 35 kg/m^2^, age > 50, neck circumference in men > 43 cm, neck circumference in women > 41 cm, and male gender). The questionnaire form was found to have a sensitivity of 83.9%, 92.9%, and 100% for detecting OSA using a cut-off score of ≥3; the specificity was 47%, 29%, and 27%, respectively. ((Apnea–Hypopnea Index (AHI) ≥5 events per hour), moderate-to-severe OSA (AHI ≥15 events per hour), and severe OSA (AHI ≥30 events per hour), respectively). The area under the curve for mild, moderate-to-severe, and severe OSA was determined to be 0.84, 0.67, and 0.63, respectively [7,23].

### 2.4. Integrated Pulmonary Index Monitoring

In EBUS procedures, all patients are typically monitored using a pulse oximeter, electrocardiogram, non-invasive blood pressure, and respiratory rate. In our study, patients undergoing the procedure under sedation were monitored using the IPI, an alternative monitor. While each of the following values—etCO_2_, respiratory rate, heart rate, and peripheral SpO_2_—was monitored separately using a capnograph device, a single numerical value indicating respiratory status, defined as the IPI, which indicates respiratory status, was obtained from the simultaneous mathematical analysis of all these parameters [16]. A special mathematical model (Fuzzy Logic Mathematical Model) is used for the algorithm employed to obtain IPI values [25]. The capnography device used in the study and the nasal cannula and mouthpiece set (Medtronic™ Capnostream^®^ 35 Portable Respiratory Monitor) were used separately for each patient. IPI takes numerical values ranging from 1 to 10 and is evaluated in six categorical groups. The IPI value is evaluated on a scale ranging from 1 (respiratory failure) to 10 (normal respiration) [16].

### 2.5. Endobronchial Ultrasonography (EBUS), Sedation and Monitoring

Real-time EBUS was performed on all included patients using a convex probe EBUS bronchoscope model EU-ME2 7602092 (Olympus Ltd., Tokyo, Japan). The procedures were performed by four interventional pulmonologists (UOS, ESSP, HH, KG) with at least two years of EBUS experience. To maintain airway patency and provide oxygen support, patients were fitted with a nasal cannula and mouthpiece set (Smart CapnoLine Guardian™-Microstream^®^, Medtronic, Minneapolis, MN, USA) connected to a capnography device (Medtronic™ Capnostream^®^ 35 Portable Respiratory Monitor, MN, USA), with 6–8 L/min of O_2_ administered via the nasal cannula. Subsequently, the following parameters were monitored: baseline, at 1 min, 5 min, 10 min, 15 min, 20 min, 25 min, 30 min, 40 min, 50 min, and 60 min post-induction IPI value, simultaneous electrocardiography monitoring, heart rate, systolic blood pressure, diastolic blood pressure, respiratory rate, SpO_2_, and etCO_2_ pressure.

No medications were administered to patients prior to the procedure for premedication purposes. Prior to sedation, a local anesthetic (Locanest 10% Lidocaine Pump) spray was applied to the patients’ oropharynx. The anesthetist administered IV 0.025–0.1 mg/kg midazolam, IV 0.5–1 μg/kg fentanyl, IV 1–1.5 mg/kg lidocaine, and IV 0.5–1 mg/kg propofol to the patients [26]. The level of sedation was assessed using the Richmond Agitation–Sedation Scale (RASS). Additional doses of propofol were administered to maintain the RASS score between −3 and −4 [27].

### 2.6. Statistical Methods

Categorical data were presented as numbers and percentages, while numerical data were presented as medians (Quartile 1–Quartile 3: Q1–Q3). The normality of numerical variables was assessed using the Shapiro–Wilk test and graphical methods (Histogram, detrended Q-Q plot). Due to the lack of normal distribution, the comparison between the two groups was performed using the Mann–Whitney U test. The comparison of categorical data was performed using Pearson’s chi-square or Fisher’s exact test. The strength of the relationship was presented using the odds ratio (OR) and the associated 95% confidence interval (CI). Logistic regression analysis was performed for low IPI and hypoxia at 5, 10, 15, and 20 min, as well as for at least one time. Results were not presented for the 25th minute and beyond, because the models did not work due to the small sample size. Age, gender, presence of any comorbidity, and pack-years of smoking were included in the model as potential confounders when investigating the association with the STOP-Bang score. Model fit was assessed using the Hosmer–Lemeshow test. Analyses were performed using Statistical Packages and Solution Services (IBM SPSS, Armonk, NY, USA) version 22. The association between low IPI and hypoxia and the STOP-Bang score was modeled using a Generalized Linear Mixed Model (GLMM). This was performed using the *GAMLj3* module with JAMOVI version 2.6.45.0. Graphs were created using the *ggplot2* package with R Project (The R Foundation for Statistical Computing, Vienna, Austria) version 4.2.2. Type 1 error was set at 0.05 (two-sided).

### 2.7. Ethical Approval

This study was approved by the institutional review board of Ankara Bilkent City Hospital (approval date: 27 November 2024, approval number: TABED 2-24-691). All participants provided written informed consent, and the study was conducted in accordance with the principles of the Declaration of Helsinki.

## 3. Results

Sixty-five patients who underwent EBUS were included in the study. Thirty-three of the participants were male (55.8%), and their median age was 62.0 (Q1–Q3= 53.0–71.0). The procedure lasted a median of 30 (Q1–Q3 = 25.0–40.0) minutes. Forty-three patients (66.2%) with a STOP-Bang score of three or higher and at high risk for OSAS were identified. The median age of patients with high STOP-Bang scores was 66.0 years (Q1–Q3 = 59.0–72), while those with low scores were 53.5 years (Q1–Q3 = 36.8–62.0), and there was a significant difference in age between the groups (*p* = 0.002). In the group of patients with high STOP-Bang scores, the BMI, neck–height ratio, comorbidity, hypertension diagnosis, and ASA score were statistically significantly higher than in patients with low STOP-Bang scores (respectively; *p* = 0.001, *p* < 0.001, *p* = 0.035, *p* < 0.001, *p* < 0.001). The distributions of demographic characteristics, comorbidities, ASA scores, vital signs, and doses of medications used for sedation in patients with high and low STOP-Bang scores who underwent EBUS are presented in Table 1.

**Figure 2:**
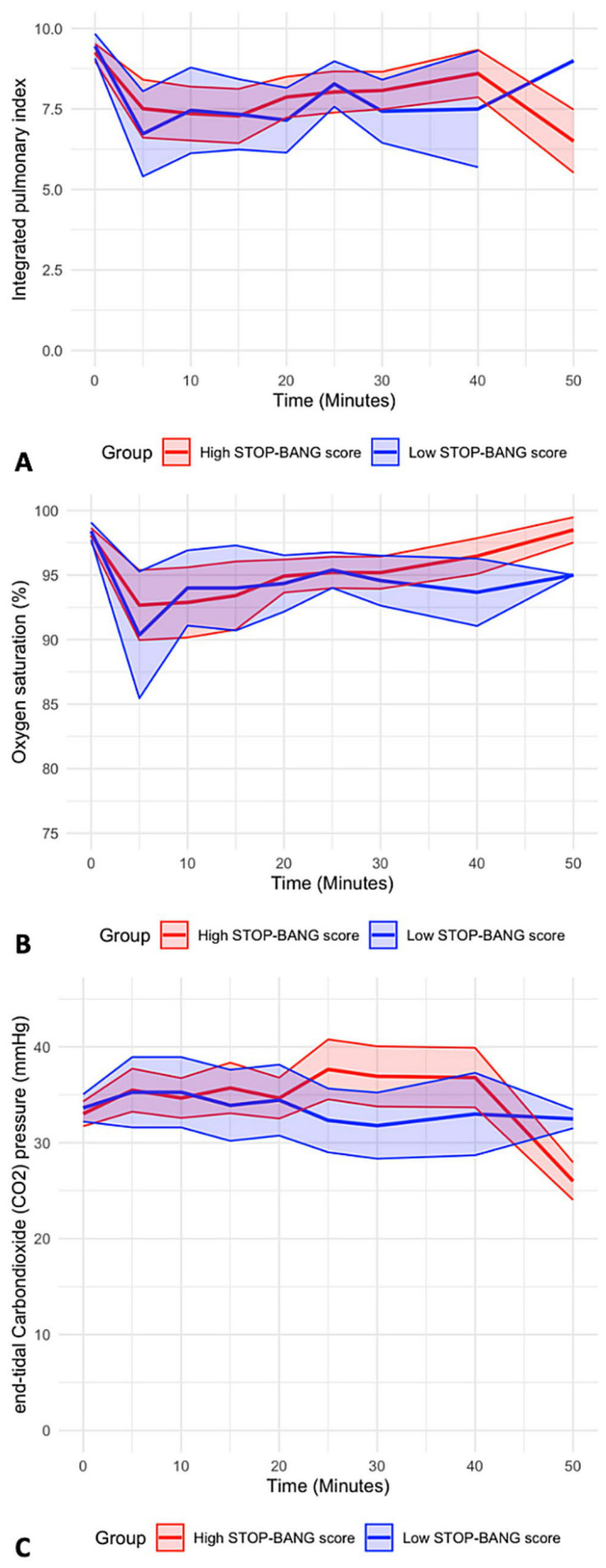
Trends over time in the mean and 95% confidence interval of the integrated pulmonary index (**A**), oxygen saturation (**B**), and end-tidal carbon dioxide pressure (**C**).

Excluding the 50th minute, where the number of patients was low, the IPI and SpO_2_ values had the lowest mean between 5 and 10 min and increased over time. The etCO_2_ pressure showed a similar trend until the 20th minute, with differences observed between the 20th and 40th minutes among the groups. Patients with high STOP-Bang scores had higher etCO_2_ pressure compared to those with low scores (Figure 2).

When factors associated with low IPI were examined at 5, 10, 15, and 20 min after the EBUS procedure, only basal IPI was associated at 5 min, while at 10, 15, and 20 min, it was associated with the previous IPI, hypoxemia status, and SpO_2_ values. No other consistently associated factors were identified throughout time. There was no statistically significant association between STOP-Bang and IPI for any of the minutes examined (Supplementary Tables S1–S4).

When patients with an IPI value below seven at least once during the EBUS procedure were compared with patients with an IPI value of seven or above, the median STOP-Bang score was 3.0 (Q1–Q3 = 2.0–4.0) in the group with a low IPI value, while the median STOP-Bang score was 4.0 (Q1–Q3 = 3.0–5.0) (*p* = 0.038). The procedure duration was statistically significantly longer in patients with low IPI values compared to those with high IPI values (*p* = 0.007). In patients who underwent EBUS, the follow-up time was found to be statistically significantly shorter in the low IPI group than in the high IPI group (*p* < 0.001). The distribution of demographic characteristics, comorbidities, ASA scores, vital signs, procedure duration, and drug doses used for the sedation of patients who had low IPI at least one time during the procedure and never had low IPI are presented in Table 2.

The interventions performed on patients during the EBUS procedure and their distribution over time are presented in Table 3. No patient’s procedure was terminated due to oxygenation problems. More interventions were performed at minutes 5 and 10 compared to other time intervals.

According to logistic regression analysis, after controlling for age, gender, pack-years of smoking, and comorbidities, the association between low IPI/hypoxia and STOP-Bang scores of 3 or higher versus scores below 3 is presented in Table 4 for minutes 5, 10, 15, 20, and at least one low IPI. According to the results of the logistic regression analysis, no statistically significant association was found between low IPI/hypoxia and either the STOP-Bang score or the STOP-Bang score being three or higher. Alternatively, findings for models including midazolam and propofol doses are presented in Supplementary Table S5.

According to the results of the GLMM regression analysis, no statistically significant association was found between high OSA risk and low IPI in individuals with a STOP-Bang score ≥ 3 compared to those with a STOP-Bang score < 3 (OR = 1.02; 95% CI = 0.36–2.86; *p* = 0.974). This analysis was adjusted for age, gender, pack-years of smoking, and comorbidities. In the GLMM regression analysis, hypoxemia in individuals with a STOP-Bang score ≥ 3 was approximately three times higher than in those with a STOP-Bang score < 3, the association was not statistically significant (OR = 3.01; 95% CI = 0.88–10.35; *p* = 0.080). Alternative GLMM models incorporating additional midazolam and propofol doses are also presented in Table 5.

## 4. Discussion

This study investigated the association between OSA risk, as assessed by the STOP-Bang questionnaire, and respiratory adverse events monitored via the IPI in patients undergoing EBUS under sedation. Although neither logistic regression nor GLMM analyses demonstrated a statistically significant association between high STOP-Bang scores and low IPI (OR = 1.02; 95% CI = 0.36–2.86; *p* = 0.974 and OR = 1.20; 95% CI = 0.45–3.22; *p* = 0.719, respectively) or hypoxemia (OR = 3.01; 95% CI = 0.88–10.35; *p* = 0.080 and OR = 2.76; 95% CI = 0.99–7.66; *p* = 0.052, respectively), patients with high STOP-Bang scores exhibited a nearly threefold higher odds of developing hypoxemia, with the GLMM result approaching statistical significance (*p* = 0.052). IPI values were consistently lowest during the 5–10-min post-induction interval in both groups, identifying a critical window of respiratory vulnerability.

The discordance between the IPI-based and hypoxemia-based outcomes warrants further discussion. Several mechanisms may explain why a high OSA risk was not reflected in IPI changes despite a near-significant association with hypoxemia. First, IPI is a composite index that integrates compensatory physiological responses, including tachycardia and increased respiratory rate; these signals may partially offset the effect of transient upper airway obstruction on the composite IPI value. Second, the IPI threshold of <7 may represent an insufficiently sensitive criterion for capturing brief desaturation events that, while meeting our operational hypoxemia definition (SpO_2_ <90% for >15 s), are not sustained long enough to substantially alter the IPI score.

Although there are no directly comparable studies examining the association between high OSA risk and low IPI in patients undergoing EBUS, it has been found that patients with high OSA risk identified by the STOP-Bang questionnaire (score ≥ 3) experienced a fourfold increase in postoperative cardiopulmonary events [28]. Similarly, patients with a STOP-Bang score ≥ 3 encounter worse perioperative respiratory outcomes and longer hospital stays [11]. These studies support the findings of our study.

A prospective study was conducted in 2024 to investigate the effect of IPI monitoring on hypoxic events compared to standard monitoring in patients undergoing EBUS under sedation. Fifty patients were included in the study. Patients were categorized into two groups: Group 1 with an IPI value of 7 or higher and Group 2 with an IPI value below 7. More apneas were detected in Group 2 compared to Group 1 (*p* < 0.05). The definition of apnea was not clearly stated in the relevant study, and apnea was detected in six (33.3%) patients in Group 2 at the 10th minute [14]. In our study, apnea was detected in only two patients at the 10th minute during the EBUS procedure. Although apnea was observed more frequently at similar times in both studies, it was considered that the lower incidence in our study may be related to the definition.

In a study published in 2018 by Ishiwata et al., which included 185 patients undergoing flexible bronchoscopy in Japan, adverse events during the procedure were compared between patients monitored with capnography and those monitored with standard monitoring techniques. Hypoxemia (SpO_2_ <90% during the procedure) was monitored using etCO_2_ monitoring without IPI assessment, and hypoxemia was more common in the standard monitoring technique group compared to the capnography-monitored patient group (*p* = 0.014) [17]. The IPI value is a value that includes etCO_2_ measured by capnography, which is a more complex and comprehensive tool for the assessment of hypoxemia and other adverse events.

In a study published in 2020, Cho et al. examined the association between the OSA risk and cardiopulmonary events that may occur during the procedure by estimating the OSA risk using the STOP-Bang questionnaire in 290 patients who underwent bronchoscopy with moderate sedation in South Korea. Hypertension was found to be more common in the group with a high STOP-Bang score (*p* < 0.001) [11]. Similarly, in our study, hypertension was found to be higher in patients with a high risk of OSA compared to the group with a low risk of OSA. When examining the association between the STOP-Bang score and cardiopulmonary events, it showed that patients with a STOP-Bang score ≥ 3 had 1.95 times more cardiopulmonary events (hypoxia and hypotension) than patients with a STOP-Bang score < 3 (OR = 1.95; 95% CI = 1.07–3.54) [11].

In a study published in 2020 involving 223 patients who underwent flexible bronchoscopy with sedation in the United States, factors associated with respiratory complications (hypoxemia (SpO_2_ ≤ 85%), bradypnea (rate), premature procedure cessation) during the procedure were examined. No association was found between the STOP-Bang score and respiratory complications [29]. In a study conducted in the United Kingdom, the STOP-Bang questionnaire was administered to 28 patients who underwent diagnostic bronchoscopy and 29 patients who underwent EBUS. Twenty-three patients were found to be at high risk for OSA. When high-risk and low-risk patients were compared, there was no statistically significant difference in the respiratory rate or SpO_2_ [30].

In our study, patients with a high STOP-Bang score were older and had a significantly higher body mass index (BMI) and neck circumference-related anthropometric measures compared with those with a low STOP-Bang score. In addition, comorbidities, particularly hypertension, were more prevalent among patients with high STOP-Bang scores.

Consistent with our findings, a prospective cohort study conducted in the United States in 2021 including 435 patients reported that participants with high STOP-Bang scores were significantly older than those with low scores (62.01 ± 16.57 vs. 56.61 ± 18.74 years; *p* = 0.01). In the same study, the BMI was higher in patients with a STOP-Bang score ≥3 compared with those with lower scores (33.93 ± 6.58 vs. 30.72 ± 5.73; *p* < 0.01), and hypertension was more prevalent in the high STOP-Bang score group (*p* < 0.01) [31].

Similarly, an analysis of data from the Korea National Health and Nutrition Examination Survey (KNHANES) 2019–2020, published in 2023 and including 7650 adults aged ≥40 years, demonstrated that male sex and hypertension were more common among individuals with high STOP-Bang scores. Moreover, age, BMI, and neck circumference values were significantly higher in the high STOP-Bang score group [32].

In our study, the neck-to-height ratio was significantly higher in patients with high STOP-Bang scores [0.24 (0.23–0.27)] compared with those with low STOP-Bang scores [0.22 (0.21–0.23); *p* < 0.001]. This finding is consistent with a study published in the United States in 2017, which included 53 patients and showed that the neck-to-height ratio was higher in patients with obstructive sleep apnea than in those without OSA (*p* = 0.04) [33].

To our knowledge, this is the first prospective study to specifically examine the association between STOP-Bang-based OSA risk stratification and IPI-derived capnographic monitoring during EBUS under sedation using a multi-timepoint design. The identification of the 5–10 min post-induction window as the period of peak respiratory compromise has direct practical implications for clinical monitoring intensity in this setting. Our study differs from other studies in the literature, as the procedure and follow-up time and, consequently, the sedation time are longer, and the study group is monitored at specific time intervals. In our study, the IPI was found to be at its lowest values in both the high-risk and low-risk OSA groups during the 5–10 min period. The dynamic trend of the IPI and other respiratory variables from the study demonstrated that cross-sectional evaluation and assessment of low IPI incidence at any given time could lead to misinterpretation. To address this issue, GLMM regression analysis was used to evaluate the association between high OSA risk and low IPI, and a statistically significant association was not determined when potential confounders were controlled. There was no statistical association between high-risk OSA and hypoxemia, although, an association close to statistical significance was identified.

This study has some limitations. First, the predictive performance of the STOP-Bang score for OSA in the study group could not be evaluated. This may have led to classification bias. Since the STOP-Bang score is known to have high sensitivity for predicting OSA [7,34], it is considered that false positive cases may have diluted the association. Although PSG remains the gold standard for OSA diagnosis, the STOP-Bang questionnaire was selected for its practical suitability in preprocedural settings: it is validated, requiring no additional equipment, cost, or specialist input. Secondly, although the specific scores of the STOP-Bang questionnaire were not disclosed to the physicians and nurses performing bronchoscopy, age, gender, and BMI, which are part of the questionnaire, may affect the administration of sedatives and the level of intervention required. Thirdly, the collection of data such as comorbidity and tobacco use based on patient reports may have caused recall bias. However, the fact that many critical data were obtained prospectively and through measurement limits errors due to recall bias. Fourth, although the fact that the data were obtained from a single center limits generalizability, the study was conducted at a reference center. Fifth, the sample size of this study may not have been sufficient to detect statistically significant associations for all outcomes of interest. The present study therefore establishes both the methodological framework and the preliminary effect size data necessary to design adequately powered future investigations into the role of OSA risk stratification and capnographic monitoring during EBUS under sedation. Lastly, because the study evaluated capnographic and oximetric changes during EBUS performed under deep sedation, obesity hypoventilation syndrome—defined by chronic daytime hypercapnia—could not be assessed or excluded. Observed etCO_2_ changes represent acute procedure-related events rather than baseline ventilatory status.

In conclusion, the observation that IPI values reach their nadir and hypoxemic episodes occur most frequently during the first 5–10 min of the EBUS procedure underscores the critical importance of heightened vigilance and prompt intervention within this vulnerable window. Although no statistically significant association was identified between high STOP-Bang scores and low IPI values—suggesting that high OSA risk, as stratified by the STOP-Bang questionnaire, may not independently predict adverse respiratory events as reflected by IPI-guided monitoring in this setting—patients with high STOP-Bang scores nonetheless demonstrated a nearly threefold higher likelihood of developing hypoxemia, a finding of considerable clinical relevance. This discordance between IPI and hypoxemia outcomes highlights the complexity of respiratory monitoring in sedated bronchoscopic procedures and suggests that OSA risk, as captured by the STOP-Bang questionnaire, may serve as a meaningful predictor of hypoxemic vulnerability even in the absence of a corresponding IPI signal. Future studies specifically designed and adequately powered to interrogate this relationship are warranted to more definitively characterize the interplay between OSA risk and adverse respiratory events during EBUS under sedation.

## Figures and Tables

**Table 1:** Distribution of demographic characteristics, comorbidities, ASA scores, vital signs, and doses of medications used for sedation in patients with high and low STOP-Bang scores who underwent EBUS.

	Total		High STOP-Bang (Score ≥ 3)		Low STOP-Bang (Score < 3)			
	n	Median (Q1–Q3) (%)	n	Median (Q1–Q3) (%)	n	Median (Q1–Q3) (%)	OR (95% CI)	*p*-Value
Age (years)	65	62.0 (53.0–71.0)	43	66.0 (59.0–72)	22	53.5 (36.8–62.0)	-	**0.002**
Male gender	33	50.8	25	58.1	8	36.4	2.43 (0.84–7.01)	0.097
BMI (kg/m^2^)	65	25.4 (22.7–29.6)	43	27.6 (24.2–31.2)	22	23.4 (20.2–25.8)	-	**0.001**
Neck–height ratio	65	0.23 (0.21–0.26)	43	0.24 (0.23–0.27)	22	0.22 (0.21–0.23)	-	**<0.001**
Comorbidities *	54	83.1	39	90.7	15	68.2	4.55 (1.16–17.82)	**0.035**
Hypertension	35	53.8	30	69.8	5	22.7	7.85 (2.36–25.81)	**<0.001**
Diabetes mellitus	14	21.5	10	23.3	4	18.2	1.36 (0.37–4.97)	0.757
COPD	6	9.2	4	9.3	2	9.1	1.03 (0.17–6.09)	>0.999
Asthma	5	7.7	5	11.6	-	-	-	0.158
CAD	14	21.5	11	25.6	3	13.6	2.18 (0.54–8.80)	0.349
CHF	5	7.7	4	9.3	1	4.5	2.15 (0.23–20.53)	0.655
Malignancy	15	23.1	11	25.6	4	18.2	1.55 (0.43–5.57)	0.503
Smoking status								0.523
Never smoker	32	49.2	19	44.2	13	59.1	1.00	
Current smoker	15	23.1	11	25.6	4	18.2	1.88 (0.49–7.22)	
Ex-smoker	18	27.7	13	30.2	5	22.7	1.78 (0.51–6.21)	
Cumulative smoking (Pack-year)	33	40.0 (22.5–60.0)	24	40.0 (30.0–60.0)	9	30.0 (15.0–70.0)	-	0.462
ASA score								**<0.001**
1	6	9.2	1	2.3	5	22.7	1.00	
2	27	41.5	14	32.6	13	59.1	5.39 (0.55–52.42)	
3	32	49.2	28	65.1	4	18.2	35.00 (3.21–381.50)	
Mallampati score								0.401
1	5	7.7	3	7.0	2	9.1	1.00	
2	38	58.5	23	53.5	15	68.2	1.02 (0.15–6.86)	
3	17	26.2	14	32.6	3	13.6	3.11 (0.35–27.54)	
4	5	7.7	3	7.0	2	9.1	1.00 (0.08–12.56)	
MAP (mmHg)	65	98.0 (90.5–107.3)	43	99.3 (93.3–105.0)	22	96.2 (86.1–108.4)	-	0.647
Pulse (/min)	65	78.0 (70.5–87.0)	43	79.0 (70.0–86.0)	22	77.5 (73.3–91.0)	-	0.551
Oxygen saturation (%)	65	97.0 (95.0–98.0)	43	97.0 (95.0–98.0)	22	97.0 (95.8–99.0)	-	0.322
Respiratory rate (/min)	65	20.0 (16.0–20.0)	43	19.0 (16.0–20.0)	22	20.0 (16.0–20.0)	-	0.639
Midazolam dose (mg)	64	2.0 (2.0–2.0)	43	2.0 (2.0–2.0)	21	2.0 (1.8–2.0)	-	0.778
Propofol dose (mg)	65	450.0 (300.0–545.0)	43	450.0 (300.0–550.0)	22	425.0 (260.0–500.0)	-	0.321
Fentanyl dose (μg)	61	100.0 (75.0–100.0)	39	100.0 (75.0–100.0)	22	100.0 (75.0–100.0)	-	0.772
Oxygen therapy (L/min)	65	12.0 (10.0–12.0)	43	12.0 (10.0–12.0)	22	11.0 (10.0–12.0)	-	0.606

OR: odds ratio, CI: confidence interval, BMI: body mass index, COPD: chronic obstructive pulmonary disease, CAD: coronary artery disease, CHF: congestive heart failure, ASA: American Society of Anesthesiologists, MAP: mean arterial pressure. * Hypothyroidism (n = 7), chronic renal failure (n = 5), hyperlipidemia (n = 4), atrial fibrillation (n = 4). Statistically significant *p*-values are shown in bold.

**Table 2:** During the EBUS procedure, at least once, patients with low IPI and patients with high IPI were compared in terms of demographics, comorbidities, ASA score, vital signs, procedure duration, and distribution of drug doses used for sedation.

	Total		Low IPI		High IPI			
	n	Median (Q1–Q3) (%)	n	Median (Q1–Q3) (%)	n	Median (Q1–Q3) (%)	OR (95% CI)	*p*-Value
Age (years)	65	62.0 (53.0–71.0)	37	62.0 (53.0–70.0)	28	61.0 (53.3–71.8)	-	0.963
Male gender	33	50.8	17	45.9	16	57.1	0.64 (0.24–1.71)	0.371
Body mass index (kg/m^2^)	65	25.4 (22.7–29.6)	37	25.4 (21.2–29.6)	28	25.5 (23.9–30.2)	-	0.292
Underweight	3	4.6	3	8.1	-	-	-	0.455
Normal	26	40.0	14	37.8	12	42.9	1.00	
Overweight	23	35.4	14	37.8	9	32.1	1.33 (0.43–4.16)	
Obesity	13	20.0	6	16.2	7	25.0	0.74 (0.19–2.79)	
Neck–height ratio	65	0.23 (0.21–0.26)	37	0.23 (0.21–0.26)	28	0.24 (0.22–0.26)	-	0.520
Comorbidities *	54	83.1	30	81.1	24	85.7	0.71 (0.19–2.73)	0.745
Hypertension	35	53.8	17	45.9	18	64.3	0.47 (0.17–1.29)	0.142
Diabetes	14	21.5	6	16.2	8	28.6	0.48 (0.15–1.60)	0.230
COPD	6	9.2	3	8.1	3	10.7	0.74 (0.14–3.95)	>0.999
Asthma	5	7.7	2	5.4	3	10.7	0.48 (0.07–3.06)	0.644
Coronary artery disease	14	21.5	10	27.0	4	14.3	2.22 (0.62–8.02)	0.216
Congestive heart failure	5	7.7	5	13.5	-	-	-	0.065
Malignancy	15	23.1	9	24.3	6	21.4	1.18 (0.36–3.81)	0.784
Smoking status								0.059
Never smoker	32	49.2	21	56.8	11	39.3	1.00	
Current smoker	15	23.1	10	27.0	5	17.9	1.05 (0.29–3.83)	
Ex-smoker	18	27.7	6	16.2	12	42.9	0.26 (0.08–0.89)	
Cumulative smoking (Pack-year)	33	40.0 (22.5–60.0)	16	40.0 (16.3–58.7)	17	40.0 (30.0–60.0)	-	0.709
ASA score								0.609
1	6	9.2	4	10.8	2	7.1	1.00	
2	27	41.5	17	45.9	10	35.7	0.85 (0.13–5.51)	
3	32	49.2	16	43.2	16	57.1	0.50 (0.08–3.13)	
Mallampati score								0.659
1	5	7.7	2	5.4	3	10.7	1.00	
2	38	58.5	22	59.5	16	57.1	2.06 (0.31–13.81)	
3	17	26.2	9	24.3	8	28.6	1.69 (0.22–12.81)	
4	5	7.7	4	10.8	1	3.6	6.00 (0.36–101.60)	
MAP (mmHg)	65	101.7 (94.5–108.5)	37	99.3 (95.5–107.3)	28	104.2 (93.8–110.8)	-	0.371
Pulse (/min)	65	79.0 (70.0–89.0)	37	83.0 (71.0–90.0)	28	78.5 (68.0–88.3)	-	0.292
Oxygen saturation (%)	65	99.0 (97.5–100.0)	37	99.0 (97.0–99.5)	28	98.5 (98.0–100.0)	-	0.930
Respiratory rate (/min)	65	18.0 (14.0–21.0)	37	18.0 (14.0–21.0)	28	16.0 (13.0–22.0)	-	0.666
etCO_2_ pressure (mmHg)	65	33.0 (30.5–36.0)	37	33.0 (30.0–35.0)	28	34.0 (32.0–37.0)	-	0.250
IPI score	65	10.0 (9.0–10.0)	37	10.0 (8.0–10.0)	28	10.0 (9.0–10.0)	-	0.799
STOP-Bang score	65	3.0 (2.0–4.0)	37	3.0 (2.0–4.0)	28	4.0 (3.0–5.0)	-	**0.038**
Low	43	66.2	21	56.8	22	78.6	0.36 (0.12–1.09)	0.066
High	22	33.8	16	43.2	6	21.4	1.00	
Midazolam dose (mg)	64	2.0 (2.0–2.0)	37	2.0 (1.0–2.0)	27	2.0 (2.0–2.0)	-	**0.041**
Propofol dose (mg)	65	450.0 (300.0–545.0)	37	450.0 (325.0–545.0)	28	420.0 (225.0–547.5)	-	0.482
Fentanyl dose (μg)	61	100.0 (75.0–100.0)	36	100.0 (75.0–100.0)	25	100.0 (75.0–100.0)	-	0.567
Oxygen therapy (L/min)	65	12.0 (10.0–12.0)	37	12.0 (10.0–12.0)	28	11.0 (10.0–12.0)	-	0.750
Operation duration (min)	65	30.0 (25.0–40.0)	37	30.0 (27.5–40.0)	28	25.0 (21.3–30.0)	-	**0.007**
Follow-up time (min)	65	15.0 (5.0–27.5)	37	10.0 (5.0–15.0)	28	25.0 (21.3–30.0)	-	**<0.001**

OR: odds ratio, CI: confidence interval, COPD: chronic obstructive pulmonary disease, ASA: American Society of Anesthesiologists, MAP: mean arterial pressure, etCO_2_: end-tidal carbon dioxide, IPI: integrated pulmonary index. * Hypothyroidism (n = 7), chronic renal failure (n = 5), hyperlipidemia (n = 4), atrial fibrillation (n = 4). Statistically significant *p*-values are shown in bold.

**Table 3:** Interventions performed on patients during the EBUS procedure and their distribution over time.

Interventions	Min 5 (n = 65) n (%)	Min 10 (n = 64) n (%)	Min 15 (n = 60) n (%)	Min 20 (n = 58) n (%)	Min 25 (n = 53) n (%)	Min 30 (n = 41) n (%)	Min 40 (n = 21) n (%)	Min 50 (n = 4) n (%)	Min 60 (n = 1) n (%)
Jaw thrust	55 (84.6)	56 (87.5)	54 (90.0)	53 (91.4)	51 (96.2)	40 (97.6)	21 (100.0)	4 (100.0)	1 (100.0)
Oropharyngeal aspiration	47 (72.3)	50 (78.1)	52 (86.7)	49 (84.5)	46 (86.8)	38 (92.7)	18 (85.7)	4 (100.0)	1 (100.0)
Increasing FiO_2_	25 (38.5)	23 (35.9)	15 (25.0)	17 (29.3)	17 (32.1)	14 (34.1)	6 (28.6)	1 (25.0)	-
Airway application	24 (36.9)	24 (37.5)	25 (41.7)	26 (44.8)	28 (52.8)	20 (48.8)	12 (57.1)	3 (75.0)	1 (100.0)
Sedation cessation	20 (30.8)	24 (37.5)	22 (36.7)	16 (27.6)	14 (26.4)	9 (22.0)	8 (38.1)	-	-
Pain stimulus	12 (13.8)	1 (1.6)	1 (1.7)	-	-	2 (4.9)	-	-	-
Interrupt the operation	9 (13.8)	2 (3.1)	2 (3.3)	-	-	-	-	-	-
Methylprednisolone administration	6 (9.2)	1 (1.6)	-	-	-	-	-	-	-

Min: minute, FiO_2_: fraction of inspired oxygen.

**Table 4:** The association between low integrated pulmonary index/hypoxia and STOP-Bang score/high obstructive sleep apnea risk according to the STOP-Bang score using the logistic regression analysis.

	**Low IPI**									
	**Min 5**		**Min 10**		**Min 15**		**Min 20**		**Ever**	
	**OR (95% CI)**	***p*** **-value**	**OR (95% CI)**	***p*** **-value**	**OR (95% CI)**	***p*** **-value**	**OR (95% CI)**	***p*** **-value**	**OR (95% CI)**	***p*** **-value**
STOP-Bang score	0.81 (0.52–1.26)	0.353	1.13 (0.74–1.73)	0.573	1.07 (0.71–1.61)	0.754	0.91 (0.57–1.46)	0.704	0.71 (0.48–1.05)	0.088
STOP-Bang score ≥ 3	1.11 (0.26–4.67)	0.891	2.85 (0.57–14.13)	0.200	2.04 (0.48–8.74)	0.335	0.66 (0.15–2.86)	0.576	0.33 (0.09–1.20)	0.093
	**Hypoxemia**									
	**Min 5**		**Min 10**		**Min 15**		**Min 20**		**Overall**	
	**OR (95% CI)**	***p*** **-value**	**OR (95% CI)**	***p*** **-value**	**OR (95% CI)**	***p*** **-value**	**OR (95% CI)**	***p*** **-value**	**OR (95% CI)**	***p*** **-value**
STOP-Bang score	1.29 (0.88–1.89)	0.200	1.20 (0.82–1.75)	0.355	1.54 (0.99–2.41)	0.058	1.21 (0.76–1.93)	0.411	1.16 (0.77–1.76)	0.478
STOP-Bang score ≥ 3	1.95 (0.58–6.55)	0.282	2.35 (0.62–8.91)	0.208	8.71 (1.46–51.88)	0.017	1.97 (0.38–10.23)	0.419	3.43 (0.90–13.05)	0.070

Adjusted for age, gender, cumulative smoking (pack-year), and presence of any comorbidity. Min: minute, OR: odds ratio, CI: confidence interval, IPI: integrated pulmonary index.

**Table 5:** The association between STOP-Bang score and low integrated pulmonary index and hypoxemia using the Generalized Linear Mixed Model regression analysis.

	OR	95% CI	*p*-Value
Low IPI			
* STOP-Bang score ≥ 3	1.02	0.36–2.86	0.974
** STOP-Bang score ≥ 3	1.20	0.45–3.22	0.719
Hypoxemia			
* STOP-Bang score ≥ 3	3.01	0.88–10.35	0.080
** STOP-Bang score ≥ 3	2.76	0.99–7.66	0.052

* Adjusted for age, gender, cumulative smoking (pack-year), and presence of any comorbidity. ** Adjusted for age, gender, cumulative smoking (pack-year), presence of any comorbidity, midazolam, and propofol dose. OR: odds ratio, CI: confidence interval, IPI: integrated pulmonary index.

## Data Availability

The datasets used and/or analyzed during the current study are available from the corresponding author on reasonable request.

## Supplementary Materials

The following supporting information can be downloaded at: https://www.mdpi.com/article/10.3390/medicina62061034/s1, Table S1: Distribution of demographic characteristics, comorbidities, ASA scores, vital signs, procedure duration, and doses of medications used for sedation in patients with low IPI and high IPI at 5 min during the EBUS procedure; Table S2: Distribution of demographic characteristics, comorbidities, ASA scores, vital signs, procedure duration, and doses of medications used for sedation in patients with low IPI and high IPI at 10 min during the EBUS procedure; Table S3: Distribution of demographic characteristics, comorbidities, ASA scores, vital signs, procedure duration, and doses of medications used for sedation in patients with low IPI and high IPI at 15 min during the EBUS procedure; Table S4: Distribution of demographic characteristics, comorbidities, ASA scores, vital signs, procedure duration, and doses of medications used for sedation in patients with low IPI and high IPI at 20 min during the EBUS procedure; Table S5: The association between low integrated pulmonary index/hypoxia and STOP-Bang score/high obstructive sleep apnea risk according to the STOP-Bang score using the logistic regression analysis.

## Abbreviations

AASM	American Academy of Sleep Medicine
AHI	apnea hypopnea index
ASA	American Society of Anesthesiologists
AUC	area under the curve
BMI	body mass index
CI	confidence interval
EBUS	endobronchial ultrasound
etCO_2_	end-tidal carbon dioxide
FiO_2_	fraction of inspired oxygen
GLMM	generalized linear mixed model
IPI	integrated pulmonary index
OR	odds ratio
OSA	obstructive sleep apnea
Q	quartile
RASS	Richmond Agitation–Sedation Scale
SpO_2_	oxygen saturation
