# Supplementary material for: The Association Between the STOP-Bang Score and the Integrated Pulmonary Index in Patients Undergoing Endobronchial Ultrasound with Sedation: The STOP OSA-IPI Cohort Study
Source: Medicina (Kaunas). 2026 May 26;62(6):1034. doi: 10.3390/medicina62061034 (PMC13303816; doi:10.3390/medicina62061034)
Supplement: Supplementary file 1 [file medicina-62-01034-s001.zip › medicina-4257192-supplementary.pdf]

Supplementary Table S1. Distribution of demographic characteristics, comorbidities, ASA scores, vital signs, procedure duration, and doses of medications used for sedation in patients with low IPI and high IPI at 5 minutes during the EBUS procedure

	n	Total<br>Median (Q1 – Q3)<br>(%)	n	Low IPI<br>Median (Q1 – Q3)<br>(%)	n	High IPI<br>Median (Q1 – Q3)<br>(%)	OR (95% CI)	p-value
Age (years)	65	62.0 (53.0 – 71.0)	18	63.0 (50.5 – 72.3)	47	62.0 (53.0 – 71.0)	-	0.883
Male gender	33	50.8	6	33.3	27	57.4	0.38 (0.12 – 1.16)	0.082
Body mass index (kg/m^2)	65	25.4 (22.7 – 29.6)	18	25.7 (22.9 – 29.5)	47	25.4 (21.6 – 29.8)	-	0.792
Neck-height ratio	65	0.23 (0.21 – 0.26)	18	0.23 (0.22 – 0.26)	47	0.23 (0.21 – 0.26)	-	0.714
Comorbidities*	54	83.1	13	72.2	41	87.2	0.38 (0.10 – 1.46)	0.161
Hypertension	35	53.8	8	44.4	27	57.4	0.59 (0.20 – 1.77)	0.347
Diabetes	14	21.5	1	5.6	13	27.7	0.15 (0.02 – 1.28)	0.089
COPD	6	9.2	-	-	6	12.8	-	0.175
Asthma	5	7.7	1	5.6	4	8.5	0.63 (0.07 – 6.07)	>0.999
Coronary artery disease	14	21.5	1	5.6	13	27.7	0.15 (0.02 – 1.28)	0.089
Congestive heart failure	5	7.7	-	-	5	10.6	-	0.311
Malignancy	15	23.1	5	27.8	10	21.3	1.42 (0.41 – 4.95)	0.743
Smoking status								**0.010**
Never smoker	32	49.2	14	77.8	18	38.3	1.00	
Current smoker	15	23.1	3	16.7	12	25.5	0.32 (0.08 – 1.36)	
Ex-smoker	18	27.7	1	5.6	17	36.2	0.08 (0.01 – 0.64)	
Cumulative smoking (Pack-year)	33	40.0 (22.5 – 60.0)	4	30.0 (20.0 – 51.3)	29	40.0 (27.5 – 60.0)	-	0.576
ASA score								0.172
1	6	9.2	3	16.7	3	6.4	1.00	
2	27	41.5	9	50.0	18	38.3	0.50 (0.08 – 2.99)	

3	32	49.2	6	33.3	26	55.3	0.23 (0.04 – 1.44)	
Mallampati score								0.371
1	5	7.7	-	-	5	10.6	-	
2	38	58.6	10	55.6	28	59.6	-	
3	17	26.2	7	38.9	10	21.3	-	
4	5	7.7	1	5.6	4	8.5	-	
MAP (mmHg)	65	101.7 (94.5 – 108.5)	18	99.3 (94.3 – 108.9)	47	102.0 (94.0 – 108.7)	-	0.901
Pulse (/min)	65	79.0 (70.0 – 89.0)	18	81.0 (69.5 – 90.3)	47	79.0 (70.0 – 89.0)	-	0.814
Oxygen saturation (%)	65	99.0 (97.5 – 100.0)	18	99.0 (97.8 – 100.0)	47	99.0 (97.0 – 99.0)	-	0.448
Respiratory rate (/min)	65	18.0 (14.0 – 21.0)	18	17.5 (14.0 – 19.3)	47	19.0 (14.0 – 22.0)	-	0.617
$etCO_2$ pressure (mmHg)	65	33.0 (30.5 – 36.0)	18	32.5 (30.8 – 36.3)	47	33.0 (30.0 – 36.0)	-	0.791
Baseline IPI	65	10.0 (9.0 – 10.0)	18	10.0 (10.0 – 10.0)	47	9.0 (8.0 – 10.0)	-	**0.010**
Hypoxemia	1	2.1	-	-	1	2.1	-	
Midazolam dose (mg)	64	2.0 (2.0 – 2.0)	18	2.0 (1.0 – 2.0)	46	2.0 (2.0 – 2.0)	-	0.197
Propofol dose (mg)	65	450.0 (300.0 – 545.0)	18	450.0 (295.0 – 542.5)	47	450.0 (300.0 – 550.0)	-	0.675
Fentanyl dose (μg)	61	100.0 (75.0 – 100.0)	17	100.0 (50.0 – 100.0)	44	100.0 (75.0 – 100.0)	-	0.339
Oxygen therapy (L/min)	65	12.0 (10.0 – 12.0)	18	12.0 (10.0 – 12.0)	47	12.0 (10.0 – 12.0)	-	0.442
STOP-Bang score	65	3.0 (2.0 – 4.0)	47	3.0 (1.8 – 4.0)	18	4.0 (2.0 – 5.0)	-	0.223
High	43	66.2	11	61.1	32	68.1	0.74 (0.24 – 2.28)	0.595
Low	22	33.8	7	38.9	15	31.9	1.00	

OR: Odds ratio, CI: Confidence interval, COPD: Chronic obstructive pulmonary disease, ASA: American Society of Anesthesiologists, MAP: Mean arterial pressure, $etCO_2$: end-tidal carbon dioxide, IPI: integrated pulmonary index

* Hypothyroidism (n=7), Chronic renal failure (n=5), Hyperlipidemia (n=4), Atrial fibrillation (n=4)

Statistically significant *p*-values are shown in bold.

Supplementary Table S2. Distribution of demographic characteristics, comorbidities, ASA scores, vital signs, procedure duration, and doses of medications used for sedation in patients with low IPI and high IPI at 10 minutes during the EBUS procedure

	n	Total Median (Q1 – Q3) (%)	n	Low IPI Median (Q1 – Q3) (%)	n	High IPI Median (Q1 – Q3) (%)	OR (95% CI)	p-value
Age (years)	64	62.0 (53.0 – 71.0)	16	61.0 (51.5 – 71.3)	48	62.0 (53.3 – 71.0)	-	0.658
Male gender	33	51.6	5	31.3	28	58.3	0.33 (0.10 – 1.08)	0.060
Body mass index (kg/m^2)	64	25.4 (22.5 -29.7)	16	27.9 (22.1 – 29.7)	48	25.3 (22.5 – 29.4)	-	0.577
Neck-height ratio	64	0.23 (0.22 – 0.26)	16	0.24 (0.22 – 0.26)	48	0.23 (0.21 – 0.26)	-	0.858
Comorbidities*	53	82.8	12	75.0	41	85.4	0.51 (0.13 – 2.05)	0.445
Hypertension	34	53.1	8	50.0	26	54.2	0.85 (0.27 – 2.63)	0.772
Diabetes	13	20.3	-	-	13	27.1	-	**0.027**
COPD	6	9.4	2	12.5	4	8.3	1.57 (0.26 – 9.51)	0.635
Asthma	5	7.8	1	6.3	4	8.3	0.73 (0.08 – 7.09)	>0.999
Coronary artery disease	14	21.9	3	18.8	11	22.9	0.78 (0.19 – 3.23)	>0.999
Congestive heart failure	5	7.8	1	6.3	4	8.3	0.73 (0.08 – 7.09)	>0.999
Malignancy	15	23.4	4	25.0	11	22.9	1.12 (0.30 – 4.18)	>0.999
Smoking status								**0.005**
Never smoker	32	50.0	12	75.0	20	41.7	1.00	
Current smoker	14	21.9	4	25.0	10	20.8	0.67 (0.17 – 2.60)	
Ex-smoker	18	28.1	-	-	18	37.5	-	
Cumulative smoking (Pack-year)	32	40.0 (21.3 – 60.0)	4	47.5 (40.0 – 79.4)	28	37.5 (20.0 – 60.0)	-	0.230
ASA score								0.626
1	6	9.4	2	12.5	4	8.3	1.00	
2	27	42.2	8	50.0	19	39.6	0.84 (0.13 – 5.56)	
3	31	48.4	6	37.5	25	52.1	0.48 (0.07 – 3.26)	

Mallampati score								0.770
1	5	7.8	-	-	5	10.4	-	
2	38	59.4	11	68.8	27	56.3	-	
3	16	25.0	4	25.0	12	25.0	-	
4	5	7.8	1	6.3	4	8.3	-	
MAP at minute 5 (mmHg)	64	103.7 (88.8 – 119.3)	16	106.5 (91.3 – 117.7)	48	102.8 (88.3 – 119.3)	-	0.816
Pulse at minute 5 (/min)	64	93.0 (77.3 – 102.8)	16	100.0 (80.8 – 109.8)	48	90.0 (73.8 – 101.5)	-	0.119
Oxygen saturation at minute 5 (%)	64	95.0 (89.3 – 98.0)	16	86.0 (77.3 – 92.0)	48	97.0 (93.3 – 99.0)	-	**<0.001**
Respiratory rate at minute 5 (/min)	64	17.0 (13.0 – 20.0)	16	16.5 (10.5 – 21.0)	48	17.0 (13.5 – 20.0)	-	0.658
$etCO_2$ pressure at minute 5(mmHg)	64	35.0 (30.3 – 39.0)	16	34.0 (31.3 – 46.3)	48	35.0 (30.0 – 39.0)	-	0.456
IPI score at minute 5	64	8.0 (6.0 -10.0)	16	3.0 (1.0 – 7.8)	48	9.0 (8.0 – 10.0)	-	**<0.001**
Hypoxemia	18	28.1	11	68.8	7	14.6	12.87 (3.42 – 48.56	0.774
Midazolam dose (mg)	37	57.8	13	81.3	24	50.0	4.33 (1.09 – 17.17)	**0.028**
Propofol dose (mg)	17	26.6	9	56.3	8	16.7	6.43 (1.85 – 22.34)	**0.007**
Fentanyl dose (μg)	63	2.0 (2.0 – 2.0)	16	2.0 (1.0 – 2.0)	47	2.0 (2.0 – 2.0)	-	0.092
Oxygen therapy (L/min)	64	450.0 (300.0 – 547.5)	16	460.0 (350.0 – 547.5)	48	450.0 (285.0 – 547.5)	-	0.451
STOP-Bang score	60	100.0 (75.0 – 100.0)	16	100.0 (50.0 – 100.0)	44	100.0 (75.0 – 100.0)	-	0.381
High	64	12.0 (10.0 – 12.0)	16	12.0 (10.0 – 12.0)	48	12.0 (10.0 – 12.0)	-	0.551
Low	64	3.0 (2.0 – 4.0)	16	3.0 (2.0 – 4.0)	48	3.5 (2.0 – 4.8)	-	0.782

OR: Odds ratio, CI: Confidence interval, COPD: Chronic obstructive pulmonary disease, ASA: American Society of Anesthesiologists, MAP: Mean arterial pressure, etCO2: end-tidal carbon dioxide, IPI: integrated pulmonary index

* Hypothyroidism (n=7), Chronic renal failure (n=5), Hyperlipidemia (n=4), Atrial fibrillation (n=4)

Statistically significant *p*-values are shown in bold.

Supplementary Table S3. Distribution of demographic characteristics, comorbidities, ASA scores, vital signs, procedure duration, and doses of medications used for sedation in patients with low IPI and high IPI at 15 minutes during the EBUS procedure

	n	Total Median (Q1 – Q3) (%)	n	Low IPI Median (Q1 – Q3) (%)	n	High IPI Median (Q1 – Q3) (%)	OR (95% CI)	p-value
Age (years)	60	61.5 (53.0 – 71.0)	19	65.0 (53.0 – 69.0)	41	61.0 (53.0 – 71.5)	-	0.987
Male gender	32	53.3	8	42.1	24	58.5	0.52 (0.17 – 1.55)	0.235
Body mass index (kg/m^2)	60	25.4 (22.6 – 30.0)	19	24.6 (20.8 – 29.8)	41	25.4 (23.7 – 29.6)	-	0.317
Neck-height ratio	60	0.23 (0.22 – 0.25)	19	0.22 (0.21 – 0.26)	41	0.23 (0.22 – 0.25)	-	0.373
Comorbidities*	49	81.7	14	73.7	35	85.4	0.48 (0.13 – 1.83)	0.277
Hypertension	30	50.0	9	47.4	21	51.2	0.86 (0.29 – 2.55)	0.781
Diabetes	13	21.7	1	5.3	12	29.3	0.13 (0.02 – 1.12)	**0.045**
COPD	6	10.0	2	15.8	3	7.3	2.38 (0.44 – 13.05)	0.370
Asthma	3	5.0	1	5.3	2	4.9	1.08 (0.09 – 12.74)	>0.999
Coronary artery disease	13	21.7	6	31.6	7	17.1	2.24 (0.63 – 7.93)	0.312
Congestive heart failure	5	8.3	3	15.8	2	4.9	3.66 (0.56 – 24.00)	0.314
Malignancy	14	23.3	4	21.1	10	24.4	0.83 (0.22 – 3.07)	>0.999
Smoking status								0.529
Never smoker	29	48.3	11	57.9	18	43.9	1.00	
Current smoker	13	21.7	4	21.1	9	22.0	0.73 (0.18 – 2.94)	
Ex-smoker	18	30.0	4	21.1	14	34.1	0.47 (0.12 – 1.79)	
Cumulative smoking (Pack-year)	31	40.0 (20.0 – 60.0)	8	35.0 (15.0 – 58.8)	23	40.0 (25.0 – 60.0)	-	0.740
ASA score								
1	6	10.0	3	15.8	3	7.3	1.00	
2	25	41.7	8	42.1	17	41.5	0.47 (0.08 – 2.87)	
3	29	48.3	8	42.1	21	51.2	0.38 (0.06 – 2.29)	

Mallampati score								0.401
1	5	8.3	-	-	5	12.2	-	
2	34	56.7	13	68.4	21	51.2	-	
3	16	26.7	5	26.3	11	26.8	-	
4	5	8.3	1	5.3	4	9.8	-	
MAP at minute 10 (mmHg)	60	93.8 (82.1 – 107.5)	19	95.0 (86.3 – 112.7)	41	92.0 (79.8 – 104.8)	-	0.291
Pulse at minute 10 (/min)	60	89.5 (76.8 – 100.0)	19	96.0 (75.0 – 103.0)	41	88.0 (77.5 – 99.5)	-	0.464
Oxygen saturation at minute 10 (%)	60	96.0 (90.5 – 98.8)	19	90.0 (85.0 – 96.0)	41	98.0 (95.0 – 99.0)	-	**<0.001**
Respiratory rate at minute 10 (/min)	60	17.0 (13.0 – 20.0)	19	15.0 (12.0 – 19.0)	41	17.0 (13.0 – 21.0)	-	0.180
$etCO_2$ at minute 10(mmHg)	60	33.5 (30.0 – 38.0)	19	37.0 (32.0 – 42.0)	41	33.0 (30.0 – 38.0)	-	0.126
IPI score at minute 10	60	8.0 (5.0 – 10.0)	19	5.0 (2.0 – 8.0)	41	9.0 (8.0 – 10.0)	-	**<0.001**
Hypoxemia	25	41.7	13	68.4	12	29.3	5.24 (1.61 – 17.01)	**0.004**
Hypoventilation	10	16.7	7	36.8	3	7.3	7.39 (1.65 – 33.12)	**0.008**
Apnea	2	3.3	-	-	2	4.9	-	-
Midazolam dose (mg)	60	2.0 (2.0 – 2.0)	19	2.0 (1.0 – 2.0)	41	2.0 (2.0 – 2.0)	-	**0.011**
Propofol dose (mg)	60	450.0 (350.0 – 550.0)	19	450.0 (350.0 – 590.0)	41	450.0 (325.0 – 550.0)	-	0.981
Fentanyl dose (µg)	56	100.0 (75.0 – 100.0)	19	100.0 (50.0 – 100.0)	37	100.0 (75.0 – 100.0)	-	0.321
Oxygen therapy (L/min)	60	12.0 (10.0 – 12.0	19	12.0 (10.0 – 12.0)	41	12.0 (10.0 – 12.0)	-	0.924
STOP-Bang score	60	3.0 (2.0 – 4.0)	19	3.0 (2.0 – 4.0)	41	3.0 (2.0 – 4.0)	-	0.733
High	39	65.0	13	68.4	26	63.4	1.25 (0.39 – 3.98)	0.705
Low	21	35.0	6	31.6	15	36.6	1.00	

OR: Odds ratio, CI: Confidence interval, COPD: Chronic obstructive pulmonary disease, ASA: American Society of Anesthesiologists, MAP: Mean arterial pressure, $etCO_2$: end-tidal carbon dioxide, IPI: integrated pulmonary index

* Hypothyroidism (n=7), Chronic renal failure (n=5), Hyperlipidemia (n=4), Atrial fibrillation (n=4)

Statistically significant *p*-values are shown in bold.

Supplementary Table S4. Distribution of demographic characteristics, comorbidities, ASA scores, vital signs, procedure duration, and doses of medications used for sedation in patients with low IPI and high IPI at 20 minutes during the EBUS procedure

	n	Total<br>Median (Q1 – Q3)<br>(%)	n	Low IPI<br>Median (Q1 – Q3)<br>(%)	n	High IPI<br>Median (Q1 – Q3)<br>(%)	OR (95% CI)	p-value
Age (years)	58	62.0 (53.0 – 71.3)	14	61.5 (53.0 – 65.55)	44	62.5 (53.3 – 72.8)	-	0.200
Male gender	30	51.7	5	35.7	25	56.8	0.42 (0.12 – 1.47)	0.169
Body mass index (kg/m^2)	58	25.4 (22.2 – 29.8)	14	22.7 (19.6 – 27.3)	44	25.9 (23.6 – 31.0)	-	0.023
Neck-height ratio	58	0.23 (0.22 – 0.26)	14	0.22 (0.21 – 0.24)	44	0.23 (0.22 – 0.26)	-	0.066
Comorbidities*	48	82.8	10	71.4	38	86.4	0.40 (0.09 – 1.67)	0.233
Hypertension	29	50.0	7	50.0	22	50.0	1.00 (0.30 – 3.33)	>0.999
Diabetes	12	20.7	2	14.3	10	22.7	0.57 (0.11 – 2.96)	0.711
COPD	6	10.3	2	14.3	4	9.1	1.67 (0.27 – 10.24)	0.624
Asthma	3	5.2	-	-	3	6.8	-	>0.999
Coronary artery disease	13	22.4	4	28.6	9	20.5	1.56 (0.40 – 6.13)	0.714
Congestive heart failure	5	8.6	3	21.4	2	4.5	5.73 (0.85 – 38.61)	0.085
Malignancy	14	24.1	1	7.1	13	29.5	0.18 (0.02 – 1.55)	0.151
Smoking status								0.272
Never smoker	28	48.3	8	57.1	20	45.5	1.00	
Current smoker	12	20.7	4	28.6	8	18.2	1.25 (0.29 – 5.35)	
Ex-smoker	18	31.0	2	14.3	16	36.4	0.31 (0.06 – 1.68)	
Cumulative smoking (Pack-year)	30	40.0 (20.0 – 60.0)	6	45.0 (37.5 – 63.1)	24	37.5 (16.3 – 60.0)	-	0.273
ASA score								0.168
1	5	8.6	3	21.4	2	4.5	1.00	
2	25	43.1	5	35.7	20	45.5	0.17 (0.02 – 1.28)	
3	28	48.3	6	42.9	22	50.0	0.18 (0.03 – 1.35)	

Mallampati score								0.127
1	5	8.6	2	14.3	3	6.8	1.00	
2	32	55.2	9	64.3	23	52.3	0.59 (0.08 – 4.12)	
3	16	27.6	1	7.1	15	34.1	0.10 (0.01 – 1.49)	
4	5	8.6	2	14.3	3	6.8	1.00 (0.08 – 12.56)	
MAP at minute 15 (mmHg)	58	113.0 (95.8 – 131.0)	14	102.5 (94.8 – 122.8)	44	116.0 (99.0 – 134.0)	-	0.148
Pulse at minute 15 (/min)	58	68.0 (58.0 – 81.0)	14	66.5 (56.0 – 74.8)	44	68.0 (60.3 – 81.0)	-	0.393
Oxygen saturation at minute 15(%)	58	82.0 (71.6 – 96.3)	14	78.7 (69.8 – 88.5)	44	84.3 (72.8 – 97.4)	-	0.203
Respiratory rate at minute 15 (/min)	58	86.0 (73.0 – 97.0)	14	89.5 (84.0 – 100.0)	44	85.5 (70.5 – 96.8)	-	0.154
$etCO_2$ pressure at minute 15 (mmHg)	58	97.0 (91.0 – 99.0)	14	88.0 (84.5 – 95.8)	44	98.0 (94.0 – 99.0)	-	0.001
IPI score at minute 15	58	17.5 (13.0 – 21.3)	14	20.0 (16.0 – 22.0)	44	17.0 (13.0 – 21.0)	-	0.228
Hypoxemia	58	33.0 (29.0 – 41.3)	14	32.0 (26.8 – 44.5)	44	33.5 (29.3 – 41.0)	-	0.636
Hypoventilation	58	8.0 (6.0 – 9.0)	14	4.5 (2.8 – 7.3)	44	8.5 (7.0 – 10.0)	-	<0.001
Apnea	19	32.8	10	71.4	9	20.5	9.72 (2.47 – 38.32)	0.001
Midazolam dose (mg)	20	34.5	9	64.3	11	25.0	5.40 (1.49 – 19.59)	0.011
Propofol dose (mg)	11	19.0	4	28.6	7	15.9	2.11 (0.52 – 8.69)	0.433
Fentanyl dose (µg)	58	2.0 (2.0 – 2.0)	14	2.0 (1.4 – 2.0)	44	2.0 (2.0 – 2.0)	-	0.265
Oxygen therapy (L/min)	58	450.0 (350.0 – 550.0)	14	375.0 (257.5 – 475.0)	44	470.0 (355.0 – 580.0)	-	0.022
STOP-Bang score	54	100.0 (75.0 – 100.0)	14	100.0 (75.0 – 100.0)	40	100.0 (75.0 – 100.0)	-	0.901
High	58	12.0 (10.0 – 12.0)	14	12.0 (10.0 – 12.0)	44	12.0 (10.0 – 12.0)	-	0.736
Low	58	3.0 (2.0 – 4.0)	14	2.5 (1.8 – 4.0)	44	3.5 (2.0 – 4.0)	-	0.207

OR: Odds ratio, CI: Confidence interval, COPD: Chronic obstructive pulmonary disease, ASA: American Society of Anesthesiologists, MAP: Mean arterial pressure, $etCO_2$: end-tidal carbon dioxide, IPI: integrated pulmonary index

*Hypothyroidism (n=7), Chronic renal failure (n=5), Hyperlipidemia (n=3), Atrial fibrillation (n=4)

Statistically significant *p*-values are shown in bold.

Supplementary Table S5. The association between low integrated pulmonary index / hypoxia and STOP-Bang score / high obstructive sleep apnea risk according to the STOP-Bang score using the logistic regression analysis

	**Low IPI**									
	**Min 5**		**Min 10**		**Min 15**		**Min 20**		**Ever**	
	**OR (95% CI)**	**p-value**	**OR (95% CI)**	**p-value**	**OR (95% CI)**	**p-value**	**OR (95% CI)**	**p-value**	**OR (95% CI)**	**p-value**
STOP-Bang score	0.80 (0.50 – 1.26)	0.331	1.15 (0.74 – 1.80)	0.530	1.18 (0.75 – 1.85)	0.471	1.03 (0.62 – 1.71)	0.904	0.66 (0.43 – 1.02)	0.061
STOP-Bang score ≥ 3	1.05 (0.23 – 4.84)	0.954	3.94 (0.60 – 25.99)	0.155	3.97 (0.70 – 22.41)	0.119	1.01 (0.21 – 4.83)	0.993	0.27 (0.07 – 1.14)	0.075
	**Hypoxemia**									
	**Min 5**		**Min 10**		**Min 15**		**Min 20**		**Overall**	
	**OR (95% CI)**	**p-value**	**OR (95% CI)**	**p-value**	**OR (95% CI)**	**p-value**	**OR (95% CI)**	**p-value**	**OR (95% CI)**	**p-value**
STOP-Bang score	1.26 (0.85 – 1.86)	0.243	1.17 (0.80 – 1.72)	0.421	1.65 (1.02 – 2.67)	0.040	1.23 (0.77 – 1.97)	0.398	1.29 (0.82 – 2.03)	0.280
STOP-Bang score ≥ 3	1.79 (0.51 – 6.32)	0.363	2.20 (0.55 – 8.74)	0.265	14.63 (2.02 – 105.87)	0.008	2.31 (0.41 – 13.16)	0.347	3.54 (0.83 – 15.21)	0.089

Adjusted for age, gender, cumulative smoking (pack-year), presence of any comorbidity, midazolam, and propofol dose

Min: Minute, OR: Odds ratio, CI: Confidence interval, IPI: integrated pulmonary index
